# MENOPAUSE HISTORY AND COGNITIVE FUNCTION IN THE IGNITE TRIAL

**DOI:** 10.1093/geroni/igae098.2011

**Published:** 2024-12-31

**Authors:** Amber Watts, Shannon Donofry, Hayley Ripperger, Nicole Eklund, Lu Wan, Chaeryon Kang, Kirk Erickson

**Affiliations:** University of Kansas, Lawrence, Kansas, United States; RAND Corporation, Pittsburgh, Pennsylvania, United States; University of Pittsburgh, Pittsburgh, Pennsylvania, United States; Boston University, Boston, Massachusetts, United States; University of Pittsburgh, Pittsburgh, Pennsylvania, United States; University of Pittsburgh, Pittsburgh, Pennsylvania, United States; Advent Health, Orlando, Florida, United States

## Abstract

Lifetime exposure to estrogen, a neuroprotectant, is associated with cognitive aging and dementia. Surgically induced menopause (ovariectomy) is associated with poorer cognitive performance, while use of hormone therapy (e.g., birth control, menopausal hormone therapy) has shown mixed associations with cognitive performance. We investigated these associations in IGNITE, a 12-month, multi-site, randomized exercise trial. We measured cognition via the Montreal Cognitive Assessment (MoCA), and factor analysis-derived composite scores for episodic memory, processing speed, working memory, attentional control, and visuospatial processing. In 461 female participants, we estimated relationships between ovariectomy, hormone-use, and baseline cognitive performance accounting for age, education, APOE ε4 carrier status, and race. Ovariectomy predicted worse MoCA scores (B=0.22, p=.038), but was unrelated to cognitive composite scores. African American race accounted for the largest proportion of variance in this model (R2Change=.09). Menopausal hormone therapy was associated with better performance on episodic memory (B=.109, p=.018) and working memory (B=.101, p=.021). Birth control use predicted better performance on MoCA (B=.090, p=.033), working memory (B=.094, p=.017), and attentional control (B=.099, p=.015). The association of ovariectomy with MoCA and not with other domains may indicate an association with dementia, not with typical cognitive aging. Meanwhile menopausal hormone therapy was associated with higher order cognitive performance, not the dementia screener. Birth control was associated with both. Our results validate prior evidence that lifetime exposure to estrogen is an important predictor of older women’s cognitive health and indicate further investigation is needed to clarify differential associations of menopause history and cognitive performance in African American women.

